# Neuroinvasion of the Highly Pathogenic Influenza Virus H7N1 Is Caused by Disruption of the Blood Brain Barrier in an Avian Model

**DOI:** 10.1371/journal.pone.0115138

**Published:** 2014-12-15

**Authors:** Aida J. Chaves, Júlia Vergara-Alert, Núria Busquets, Rosa Valle, Raquel Rivas, Antonio Ramis, Ayub Darji, Natàlia Majó

**Affiliations:** 1 Centre de Recerca en Sanitat Animal (CReSA), UAB-IRTA, Campus de la Universitat Autònoma de Barcelona, Barcelona, Spain; 2 Departament de Sanitat i Anatomia Animals, Universitat Autònoma de Barcelona, Barcelona, Spain; 3 Institut de Recerca i Tecnologia Agroalimentàries (IRTA), Barcelona, Spain; Washington University, United States of America

## Abstract

Influenza A virus (IAV) causes central nervous system (CNS) lesions in avian and mammalian species, including humans. However, the mechanism used by IAV to invade the brain has not been determined. In the current work, we used chickens infected with a highly pathogenic avian influenza (HPAI) virus as a model to elucidate the mechanism of entry of IAV into the brain**.** The permeability of the BBB was evaluated in fifteen-day-old H7N1-infected and non-infected chickens using three different methods: (i) detecting Evans blue (EB) extravasation into the brain, (ii) determining the leakage of the serum protein immunoglobulin Y (IgY) into the brain and (iii) assessing the stability of the tight-junction (TJ) proteins zonula occludens-1 and claudin-1 in the chicken brain at 6, 12, 18, 24, 36 and 48 hours post-inoculation (hpi). The onset of the induced viremia was evaluated by quantitative real time RT-PCR (RT-qPCR) at the same time points. Viral RNA was detected from 18 hpi onward in blood samples, whereas IAV antigen was detected at 24 hpi in brain tissue samples. EB and IgY extravasation and loss of integrity of the TJs associated with the presence of viral antigen was first observed at 36 and 48 hpi in the telencephalic pallium and cerebellum. Our data suggest that the mechanism of entry of the H7N1 HPAI into the brain includes infection of the endothelial cells at early stages (24 hpi) with subsequent disruption of the TJs of the BBB and leakage of virus and serum proteins into the adjacent neuroparenchyma.

## Introduction

Central nervous system (CNS) lesions induced by influenza viruses have been frequently described in a number of animal species, including poultry and wild birds, cats, horses and laboratory animals [1], [2], [3], [4], [5], [6]. In humans, different strains of influenza A virus (IAV) (mainly from the H1N1 and H3N2 subtypes) have also been shown to occasionally induce CNS [7], [8] lesions.

Most of the studies related to the neuropathogenicity of influenza virus have been conducted using mice, in which the virus mainly uses nervous routes to cause CNS lesions [2], [9], [10]. The mouse model has been used to study the non-purulent encephalopathies associated with influenza virus infection observed in humans. These encephalopathies, including von Economo’s encephalitis or *encephalitis lethargica* and post-encephalitic Parkinsonism, are hypothesized to occur by viral invasion of the brain through a nervous route [11]. There is a second group of human influenza-associated encephalopathies that includes necrotizing encephalopathy (ANE) of childhood [12], [13], hemorrhagic shock and encephalopathy [14], and Reye’s syndrome [15]. This group of encephalopathies, characterized by the induction of a necrotizing encephalopathy, are believed to occur through disruption of the BBB [12], but the mechanism leading to this disruption is unknown [9].

The blood brain barrier (BBB) is a neurovascular filtering system that also serves as a selective diffusion barrier that protects the brain from the entry of potentially toxic molecules and infectious agents. The BBB is composed of endothelial cells that are firmly sealed by tight junctions (TJs) and supporting cells. However, this barrier can be surmounted by different pathogens, as described for human immunodeficiency virus (HIV) [16], simian immunodeficiency virus (SIV), feline immunodeficiency virus [17], measles virus, human cytomegalovirus (HCMV), human T-cell leukemia virus (HTLV) [18] and West Nile virus [19]. These viruses have developed strategies that include: 1) passage of cell-free virus into the brain using paracellular or transcellular routes, 2) traversal of the BBB inside infected leucocytes or a “Trojan horse” mechanism, and 3) direct replication of the virus in endothelial cells or astrocytes causing BBB breakdown and entry of the virus to the brain parenchyma [20].

In a previous study, we described the topographical distribution of an H7N1 HPAI virus in the CNS at the early stages of infection. It was concluded that the virus spreads to the CNS by a hematogenous route, and it likely enters the brain after disruption of the BBB [21]. Although this fact has not been completely elucidated, our findings support the idea that the chicken can be a good animal model for understanding the mechanism underlying this group of influenza-associated necrotizing encephalopathies in humans.

The main objective of this study was to evaluate the ability of the H7N1 HPAI virus (A/Chicken/Italy/5093/99) to invade the CNS of chickens through the disrupted BBB. Three different approaches were used to investigate how this HPAI virus damages the BBB: (i) an approach based on the detection of Evans blue (EB) extravasation, (ii) an approach determining the leakage of the serum protein immunoglobulin Y (IgY) and (iii) an approach assessing the stability of the tight-junction (TJ) proteins zonula occludens-1 and claudin-1 at early post-infection stages in different brain regions. The usefulness of this model for studying influenza-associated encephalopathies was also evaluated.

## Materials and Methods

### Virus

The influenza virus used in this study was kindly provided by Dr. Moreno and corresponds to a fifth passage H7N1 HPAI virus strain A/chicken/Italy/5093/99 that possesses an intravenous pathogenicity index of 2.8. To prepare the virus for the study, virus was propagated once in 10-day-old embryonated specific pathogen free (SPF) chicken eggs. Allantoic fluid was titrated in SPF eggs according to the method of Reed and Muench [22] and later diluted in PBS to obtain a dose of 10^6^ egg lethal dose 50% (ELD_50_) in 0.05 mL (50 µL).

### Experimental design

All experiments with HPAIV were performed at Biosafety Level 3 facilities of the *Centre de Recerca en Sanitat Animal* (CReSA-Barcelona). The present study was conducted in accordance with the Guidelines of the Good Experimental Practices and under the supervision and approval of the Ethical and Animal Welfare Committee of the UAB (*Permit Number*: DMAH-4239).

**Figure 1:**
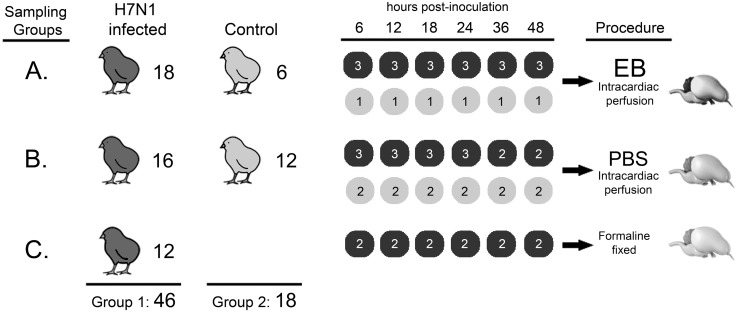
Experimental design. Procedures followed for each group of chickens included in the study. Chickens were divided in two groups; the first consisted of 46 chickens that were inoculated with 10^6^ ELD_50_ of H7N1 A/Chicken/Italy/5093/99 HPAI virus, while the second group of 18 chickens were used as non-infected control. In turn, the inoculated chickens were divided in three groups (A, B, and C), as well the non-infected chickens that were divided en two groups. Group A consists of 18 H7N1 inoculated chickens and 6 non-infected chickens that were perfused with EB at 6, 12, 18, 24, 36 and 48 hpi and their brains were frozen. Group B includes 16 H7N1 inoculated and 12 non-infected chickens that were perfused with PBS and their brains were frozen. Group C contains 12 chickens inoculated with H7N1 and which brains were fixed in formalin.

To determine the mechanism and time of disruption of the BBB, sixty-four 15-day-old SPF chickens were divided in two groups (Fig. 1). The first group (G1) consisted of 46 chickens that were intranasally inoculated with 10^6^ ELD_50_ of H7N1 A/Chicken/Italy/5093/99 HPAI virus and a second group of eighteen chickens that were used as uninfected controls. The 46 infected chickens in the first group were further subdivided into three sampling groups (A, B, C). Similarly, the 18 uninfected chickens were subdivided and used as controls for sampling groups A (six chickens) and B (12 chickens) groups. Blood samples were taken from all animals to determine the presence of viremia.

Sampling group A consisted of 18 infected chickens that were used to evaluate BBB stability via intracardial perfusion of the immunofluorescence tracer Evans blue (EB) at 6, 12, 18, 24, 36 and 48 hours post infection (hpi) (3 chickens for each hpi). Additionally, one uninfected chicken was used as a control and was also perfused and sampled at the same hpi. The perfusion was performed first using 50 mL phosphate buffered saline solution (PBS) and then 50 mL of a “cocktail” containing EB and paraformaldehyde diluted in PBS pH 7.2, prepared as previously described [23]. To perfuse the chickens, they were deeply anesthetized with 50 mg/kg of sodium pentobarbital administered intravenously. Once the chickens were unresponsive to stimulus, the coelomic cavity was immediately opened. Later, a polished 21-gauge needle was inserted through the left ventricle into the ascending aorta and a gravity-dependent perfusion system was rapidly connected to the needle. Then, an incision was made in the right atrium to drain incoming venous blood and the perfusion solution that was administered into the left ventricle. The mean flow rate was 2 mL/min. Brain samples from these chickens were carefully collected and used to determine the presence of EB and IgY extravasation.

Sampling group B consisted of sixteen infected chickens that were perfused with PBS. Similar to the previous group, perfused brain samples from three chickens were collected at 6, 12, 18, and 24 hpi, whereas at 36 and 48 hpi, two infected chickens were evaluated. Perfused brain samples from two uninfected control chickens were examined at the same hpi. Brain samples obtained from these chickens were cut in five coronal sections and immediately embedded in OCT-Fast Frozen compound (Tissue-Tek, Torrance, CA), fast-frozen using isopentane (M32631, Sigma-Aldrich Química, S.A., Madrid, Spain) in liquid nitrogen, and stored at −80°C until use. The presence of IgY extravasation and influenza A virus antigen in these samples was assessed, along with the pattern of ZO-1 and claudin-1 protein staining.

The third sampling group (C) included 12 infected non-perfused chickens, the brains of which were sampled at 6, 12, 18, 24, 36 and 48 hpi (two brains for each hpi) and fixed in 10% buffered formalin. These chicken brains were used to determine the distribution of the influenza A virus antigen.

### Processing and analysis of EB-perfused chicken brain samples

EB-perfused brain samples obtained from sample group A were postfixed in 4% paraformaldehyde (15714, Electron Microscopy Science) for 12 h and cryoprotected by immersion in 30% sucrose (84097, Sigma-Aldrich Química, S.A.) for 24 h. Afterward, brains embedded in cryostat-embedding compound (OCT-Fast Frozen compound) (Tissue-Tek, Torrance, CA) were cut in six gross coronal sections and fast frozen using isopentane (M32631, Sigma-Aldrich Química, S.A.) chilled in liquid nitrogen. Samples were stored at −80°C until used. Later, 10-µm thick cryostat (Leica Microsystems, Germany) sections were prepared at −22°C, mounted on SuperFrost plus glass slides (631–9483, VWR International Eurolab, S.L) and stored at −20°C until used. To directly evaluate the disruption of the BBB, slides of the six coronal sections from perfused infected and control chickens were fixed with acetone for five minutes (min), washed three times for five min with PBS pH 7.4, and cover-slipped using Vectashield mounting medium (H-1000, Vector Labs, Burlingame, CA). Images from these samples were captured with a spot digital camera (Nikon DXM1200F) coupled to a Nikon microscope (Nikon eclipse 90i) using Nikon ACT-1 software.

### Immunofluorescence staining for the detection of influenza A virus antigen in perfused and fresh frozen brain sections

Sequential brain sections from all infected EB- and PBS-perfused chickens, as well as brain sections from control chickens collected at 6, 12, 18, 24, 36 and 48 hpi, were cut into 10-µm thick sections, mounted on SuperFrost Plus slides and stored at −20°C until used. Later, sections were allowed to thaw for 30 min at room temperature (RT) and then fixed using 95% ethanol at 4°C for 15 min and acetone for one min at RT. Samples were then rinsed three times with PBS pH 7.4 and blocked with 2% bovine serum albumin (BSA) (85040C, Sigma-Aldrich Química, S.A.) in PBS pH 7.4 containing 0.1% Triton X-100 (T8787, Sigma-Aldrich Química, S.A.) for one h at RT. Afterwards, brain sections were incubated overnight at 4°C with an anti-nucleoprotein (NP) monoclonal antibody (ATCC, HB-65, H16L-10-4R5) diluted 1∶100 in blocking buffer. The next day, samples were rinsed with PBS and incubated for 1 h at RT with DyLight488 goat anti-mouse IgG secondary antibody (115-485-166, Jackson ImmunoResearch Lab, USA) diluted 1∶200 in PBS. Nuclear counterstaining was performed using Hoechst 33258 (Sigma-Aldrich Química, S.A.) diluted 1∶500 in PBS for 10 min at RT. Samples were rinsed with PBS and coverslipped with anti-fade Vectashield mounting medium. Negative controls consisted of sequential samples of the same tissue incubated with blocking buffer instead of the primary antibody. A positive control was also included with each batch and consisted of tissues of chicken embryos inoculated with the same H7N1 HPAIV strain. EB extravasation zones were visualized in the red channel, whereas influenza A virus (IAV) antigen was visualized in the green channel. Photomicrographs were merged using Adobe Photoshop CS2 (Adobe System Inc., San Jose, CA).

### Immunofluorescence staining for the detection of IgY extravasation in perfused brain sections

The breakdown of the BBB was also evaluated by the detection of an increase in the vascular permeability to endogenous IgY. For these experiments, 10-µm thick cryostat brain sections from two infected chickens perfused with EB and three perfused with PBS, as well as sections from control chickens, were processed as described above for the detection of the influenza A virus antigen, with some modifications. Briefly, tissue sections were blocked using 5% normal donkey serum (NDS) (D9663, Sigma-Aldrich Química, S.A.), 1% bovine serum albumin (BSA), and 0.2% Triton diluted in PBS pH 7.4 (5% NDS/1% BSA/0.1% triton). Later, the samples were incubated with fluorescein isothiocyanate (FITC)-conjugated donkey anti-chicken IgY (H+L) (DAIgY-F, Gallus Immunotech, Inc, Canada) diluted 1∶50 in the same blocking buffer. IgY extravasation was observed around the vessels in the green channel. Brain sections of mice were used as negative control to evaluate the specificity of the antibody, considering that the primary antibody was raised against a chicken IgY that differed antigenically from the mammalian IgG [24], [25].

### Double immunofluorescence staining for codetection of IgY leakage and influenza A virus antigen in fresh frozen brain sections

To determine the presence of IgY extravasation in influenza A virus-positive zones, fresh frozen brain sections from infected and control chickens collected at 18, 24, 36 and 48 hpi were processed as previously described for the detection of IgY, with some modifications. Briefly, 10-µm brain sections were incubated with a mixture of FITC-conjugated donkey anti-chicken IgY (H+L) (1∶50) and anti-NP monoclonal antibody (ATCC, HB-65, H16L-10-4R5) (1∶100) diluted 1∶100 in 5% NDS/1% BSA/0.1% Triton in a total of 200 µl per section at 4°C overnight. The next day, the samples were incubated for one h at RT with a Cy3-conjugated goat anti-mouse IgG (H+L) (115-165-003, Jackson ImmunoResearch Lab, USA) diluted 1∶200 in PBS. The non-specific binding of the Cy3-conjugated secondary antibody to the IgY antibody was tested by incubating sequential brain sections from the same chickens with the FITC-conjugated donkey anti-chicken IgY antibody alone, followed by one h of incubation with the Cy3-conjugated goat anti-mouse IgG secondary antibody.

### Immunofluorescence staining for the detection of the tight junction proteins claudin-1 and zonula occludens-1 (ZO-1) in perfused brain sections

To evaluate the effect of the virus on the BBB, the presence, distribution and pattern of staining of the BBB proteins ZO-1 and claudin-1 were tested in brain sections from all chickens perfused with PBS and control chickens subjected to the same process and obtained at the same sampling hours. For these experiments, the same protocol used for the detection of the influenza A virus NP was used to detect the ZO-1 and claudin-1 proteins, with some modifications. Briefly, 10-µm cryostat brain sections were incubated with primary antibodies at 4°C overnight: anti-ZO-1 rat monoclonal antibody (Clone R40.76, Chemicon, Tamecula, CA. USA) or anti-claudin-1 rabbit antibody (51–9000, Invitrogen, S.A.) was diluted 1∶50 in PBS containing 1% BSA and 0.1% Triton. The ZO-1 signal was visualized using DyLight 488 rabbit-anti-rat IgG secondary antibody (312-485-003, Jackson ImmunoResearch Lab, USA), whereas the claudin-1 signal was detected using FITC-conjugated goat–anti-rabbit secondary antibody (F9887, Sigma-Aldrich Química, S.A.). Both secondary antibodies were diluted 1∶200 in PBS and incubated with samples for one h at RT. Positive controls for the detection of claudin-1 were brain samples from a rat, whereas the specificity of the ZO-1 antibody was tested on brain samples from mice. Negative controls consisted of sequential sections from the experimental brain samples incubated with blocking buffer lacking primary antibody. Both signals were observed in the green channel.

### Double immunofluorescence staining for the codetection of ZO-1 and influenza A virus antigen in fresh frozen brains sections

Fresh frozen brain sections from two infected chickens and one control chicken collected at 6, 12, 18, 24, 36 and 48 hpi were processed for the codetection of ZO-1 and influenza A virus antigen. For these experiments, 10-µm cryostat sections were incubated first with the anti-ZO-1 rat monoclonal antibody diluted 1∶50 in blocking buffer (prepared as described for the detection of influenza A virus antigen) at 4°C overnight. Afterwards, the samples were incubated for one h at RT with a Cy3-conjugated goat anti-rat IgG (H+L) secondary antibody (112-165-062, Jackson ImmunoResearch Lab, USA) diluted 1∶200 in 1% BSA PBS pH 7.4. Next, the samples were blocked by incubation in 2% BSA in PBS for one h and then incubated at 4°C overnight with anti-influenza virus nucleoprotein (NP) monoclonal antibody (ATCC, HB-65, H16L-10-4R5) diluted 1∶100 in the same blocking buffer. The next day, the samples were incubated for one h at RT with a Cy2-conjugated goat anti-mouse IgG, subclass 2a (115-225-206, Jackson ImmunoResearch Lab, USA) diluted 1∶100 in PBS pH 7.4. In these experiments, the ZO-1 staining was visualized in the red channel and the influenza A virus antigen was visualized in the green channel. Negative controls consisted of incubation of sequential samples with 2% BSA in PBS without the inclusion of a primary antibody. The non-specific binding of both secondary antibodies was ruled out by incubating them with the contrary primary antibody.

### Immunohistochemical staining of influenza A virus antigen to determine the topographical distribution of the viral antigen and the initial target cells in the brain of chickens during the first hpi

Formalin-fixed brain samples were cut in six different coronal sections and later embedded in paraffin. The immunohistochemical technique used for the detection of influenza A virus NP was performed as previously described [26], [27]. Briefly, brain sections (3-µm thick) were dewaxed and treated with 3% H_2_O_2_ in methanol to eliminate endogenous peroxidase activity. Later, antigen retrieval was performed using protease at 37°C for 10 min followed by incubation at 4°C overnight with the primary monoclonal antibody (ATCC, HB-65, H16L-10-4R5) diluted 1∶250. The next day, the samples were incubated with biotinylated goat anti-mouse IgG secondary antibody (Dako, Immunoglobulins AS, Denmark) and the avidin-biotin-peroxidase complex (ABC) (Thermo Fisher Scientific, Rockford, IL, USA). The reaction was developed at RT using 3,3′-diaminobenzidine tetrahydrochloride (DAB) (Sigma-Aldrich, MO, USA), and counterstaining was performed using Mayer’s hematoxylin.

The distribution, intensity and pattern of viral antigen staining in the CNS of chickens at each hpi were evaluated and later scored as described in a previous study (8). Briefly, the following regions were evaluated in the six different coronal sections: the olfactory bulb (OB), telencephalic pallium (Pall), telencephalic subpallium (Spall) (containing the striatum (St)), hypothalamus, optic area (Och), diencephalon (containing the prethalamus (p3), thalamus (p2), pretectum (p1), and the secondary prosencephalon (2P)), midbrain or mesencephalon, hindbrain (containing the isthmus (Ist) and rhombencephalon (r1-6)), and the cerebellum (Cb). In these regions, the number of viral antigen-positive cells in 10x fields was counted. As the extension of each region was variable, an average was calculated for each region. At the same time, at 36 and 48 hpi, an arithmetic mean for each region was obtained for the two evaluated animals. The intensity of the staining was assessed using a semi-quantitative scoring: nil (0: no labeling detected); scarce (1: less than 20 nuclei of cells positive for viral antigen on average), slight (2: more than 20 but less than 100 positive cells on average). We did not rank moderate and intense viral antigen staining, because in comparison with previous studies, the number of viral antigen-positive cells was low (less than 100 positive cells per 10x field) [21]. Finally, the topographical distribution of the viral antigen staining was graphically represented in the six coronal sections using Adobe Photoshop CS2.

### Quantification of viral RNA in the blood of chickens by quantitative real time RT-PCR (RT-qPCR)

The RT-qPCR technique used here to quantify viral RNA copies in blood and brain tissue samples has been thoroughly described [28]. Briefly, viral RNA was extracted from each sample using a QIAamp Viral Mini kit (Qiagen, Hilden, Germany). The viral RNA obtained was eluted in 40 µL and tested by one-step RT-qPCR for the detection of a highly conserved region of the matrix (M1) gene of the H7N1 influenza A virus using published specific primers [29] and previously described amplification conditions [28] in a Fast7500 thermocycler (Applied Biosystems). This procedure uses an internal positive control (IPC) to avoid false negative results due to RT-PCR inhibitors.

## Results

### Clinical evaluation

No clinical signs compatible with HPAI virus infection such as depression, ruffled feathers, prostration or skin hemorrhages, were observed in either infected or control chickens. Gross lesions were not observed at any hpi.

### EB extravasation in brains from control and H7N1-inoculated chickens

**Figure 2:**
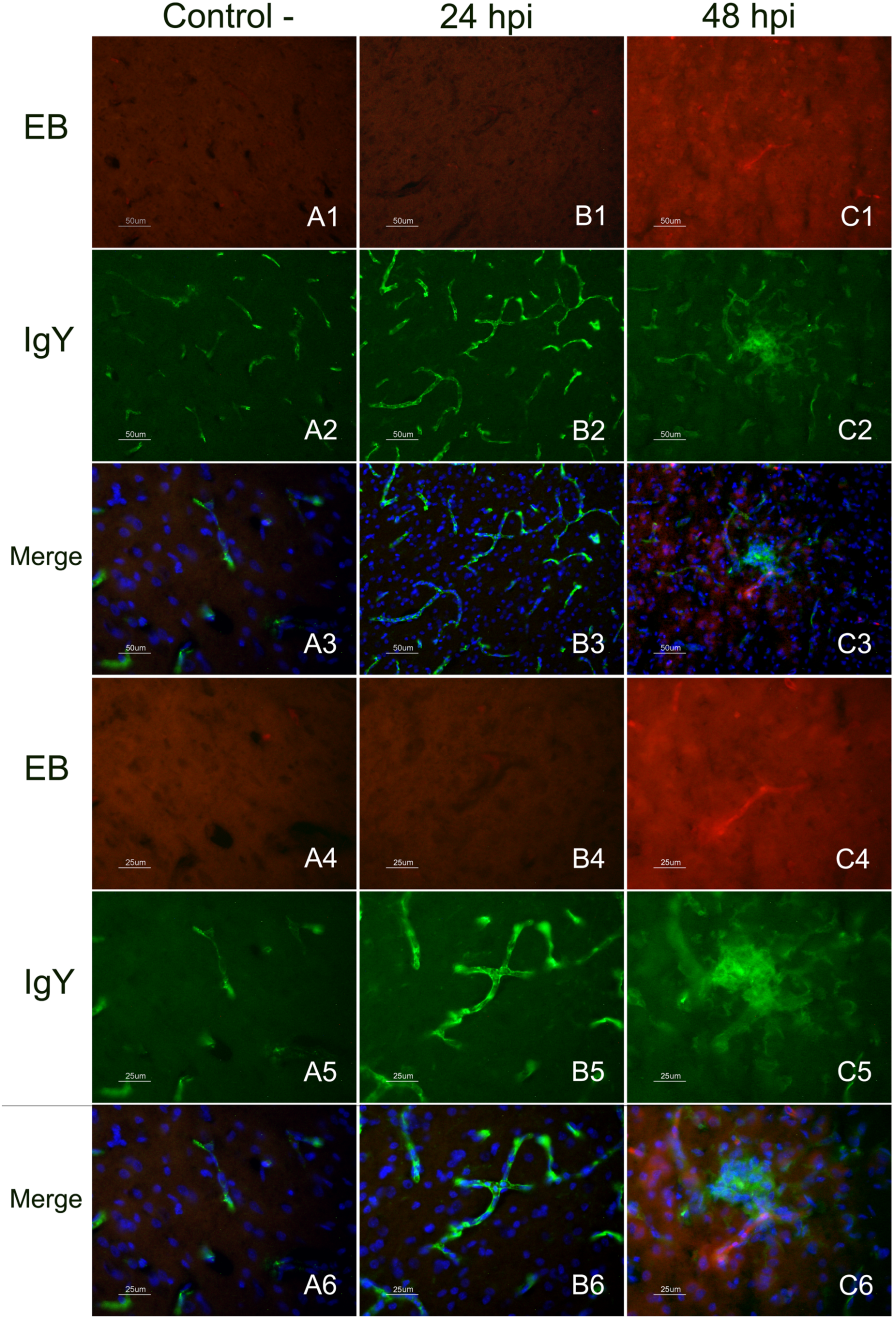
Immunofluorescence staining to detect IgY leakage in EB perfused and fresh frozen brain sections. Detection of EB and IgY extravasion in the telencephalic pallium (Pall) of infected chickens at 24 and 48 hpi in comparison with a control chicken at 48 hpi (figures in the top, from A1 to C3 measure 50 µm and figures in the bottom measure 25 µm). EB extravasation (red colour) in the telencephalic pallium (Pall) was only observed in brain samples of chickens evaluated at 48 hpi (C1, C4). Images at two different magnifications showing a microvessel with a fan-like area of EB leakage (C1, C4). No EB extravasation was observed in non-infected control chickens perfused with EB at 48 hpi (A1, A4), nor EB extravasation was detected on infected chickens at 24 hpi (B1, B4). Leakage of the serum protein IgY (C2, C5) was observed in the vessels and the nearest brain cells in infected chickens perfused at 48 hpi (green colour). IgY staining in control (A2, A5) and infected chickens at 24 hpi (B2, B5) was limited to the lumen of the vessels. Merged image allowed demonstrating the presence of colocalization of IgY leakage in areas of EB extravasation in chickens evaluated at 48 hpi (C3, C6). Controls chickens evaluated at 48 hpi and infected chickens at 24 hpi did not show EB leakage and the IgY staining was limited to the lumen of the vessels.

EB extravasation in infected animals was not detected until 48 hpi, when multiple foci of intense and bright red staining were observed. These foci corresponded to blood microvessels of different caliber, which usually showed a rim of EB leakiness or a fan shape of EB extravasation (Fig. 2-C1, C6). In these areas of EB extravasation, the neuropil and the nearest neural cells were intensely stained. These foci were widely distributed in the brains of all infected chickens, which showed these focus in the telencephalic pallium and cerebellum, followed by the thalamus (p2), Spall and brainstem. In the OB, mesencephalon and rhombencephalon, fewer foci of EB extravasation were observed. No evidence of EB leakage was observed in the brains of control chickens at any hpi. In the control chickens, the EB staining was imperceptible and restricted to the lumen of some vessels along the brain and in the meninges (Fig. 2-A1, A6). The endothelial cells in the choroid plexus also showed EB staining. Furthermore, some epithelial cells of the choroid plexus exhibited weak EB staining. The circumventricular organs (CVOs), which are brain areas normally lacking a complete BBB, including the medial eminence (ME) and area postrema (AP), showed light staining. Similarly, the brains of chickens perfused at 6, 12, 18, and 24 hpi showed only weak EB staining in blood vessels, with no diffusion to the surrounding brain tissue.

### Detection of IAV antigen in zones of EB extravasation

**Figure 3:**
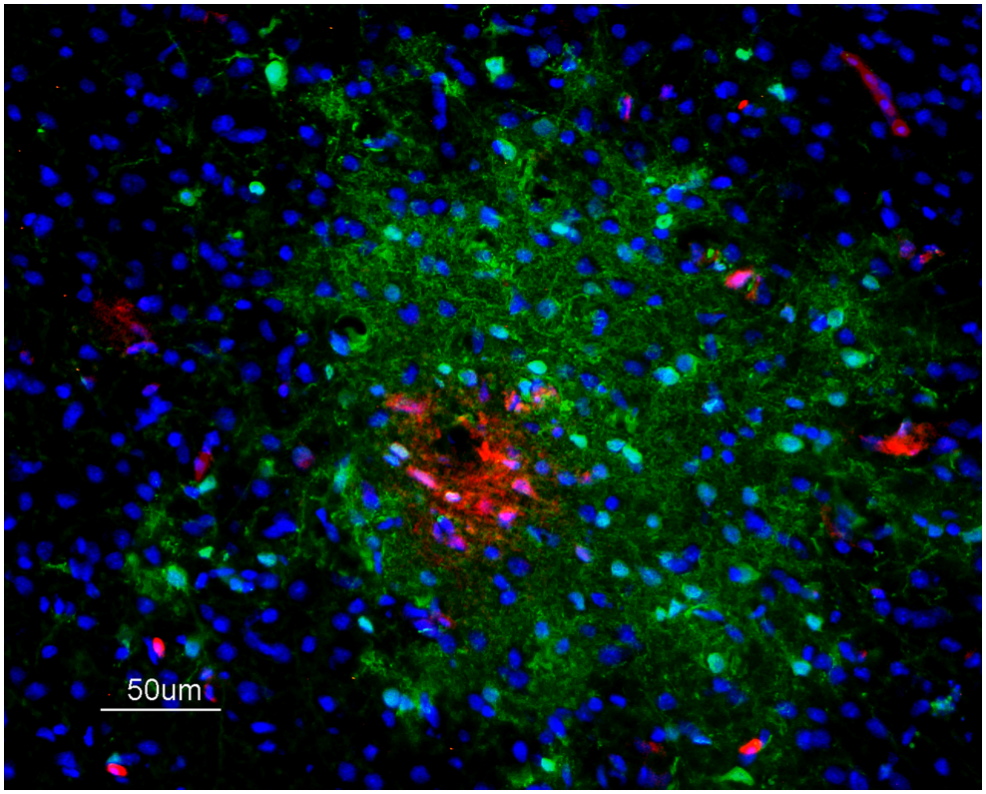
Immunofluorescence staining to detect IAV antigen in brain samples of EB-perfused H7N1 infected chickens. IAV antigen (green) labelling was codetected in areas of EB extravasation (red) and extends surrounding the leakage area in a chicken at 48 hpi, 50 µm.

IAV antigen was detected in the zones of EB extravasation at 48 hpi, but typically the IAV-positive zones were more extensive than the EB diffusion ratio (Fig. 3). It was also common to see brain microvessels showing extravasion of EB without the presence of the viral antigen. These areas of EB and viral antigen co-localization were commonly observed multifocally in the telencephalon and cerebellum. They were observed less frequently in the diencephalon, mesencephalon and rhombencephalon. In these areas, the presence of the viral antigen and EB extravasation was multifocal and, exceptionally in the *Rotundus* (Rot) nucleus of the thalamus, EB extravasation was located bilaterally in the vessels surrounding the nucleus. IAV antigen was also observed in tissue of PBS-perfused infected chickens at 24, 36 and 48 hpi. At 24 hpi, the staining was restricted to blood vessel cells. After 24 hpi, the presence of IAV antigen in endothelial cells was observed mainly in the nucleus but also in the cytoplasm. At 36 and 48 hpi, IAV antigen-positive parenchymal cells (glial cells and neurons) formed foci or were distinct positive cells. Moreover, IAV antigen was detected in the ependymal cells lining the lateral, third and fourth ventricles. The presence of viral antigen was mainly observed in the nucleus of neural and glial cells, but it was also detected in the cytoplasmic processes of these cells, as well as in the neuropil.

### Detection of IgY extravasation in perfused brain sections

The detection of IgY was used to determine the presence of extravasation of this endogenous serum protein into the extravascular space [30], [31]. IgY staining was restricted to the lumen of blood microvessels in the parenchyma of the brain, meninges and choroid plexus of control chickens (Fig. 2-A2, A5); as well as in samples from infected chickens between 6 and 24 hpi (Fig. 2-B2, B5). The CVOs were diffusely immunostained, with IgY being widely distributed in the vessels, ependymal cells and parenchyma of both healthy and infected chickens. IgY leakage was observed in infected chickens at 36 and 48 hpi. The most affected brain regions were the telencephalic pallium and cerebellum, where IgY extravasation was multifocal. In these regions, IgY extravasation was observed as a slightly stained green rim around the vessels and as focal zones of diffusion where the neuropil and nearest glial and neural cells showed green staining (Fig. 2-C2, C5). IgY extravasation increased with time, being most severe at 48 hpi. Other brain regions, such as the diencephalon and mesencephalon, showed zones of IgY extravasation; however, the extravasation in these regions was less severe and frequent.

### Colocalization of IgY extravasation and IAV in fresh frozen brain samples

**Figure 4:**
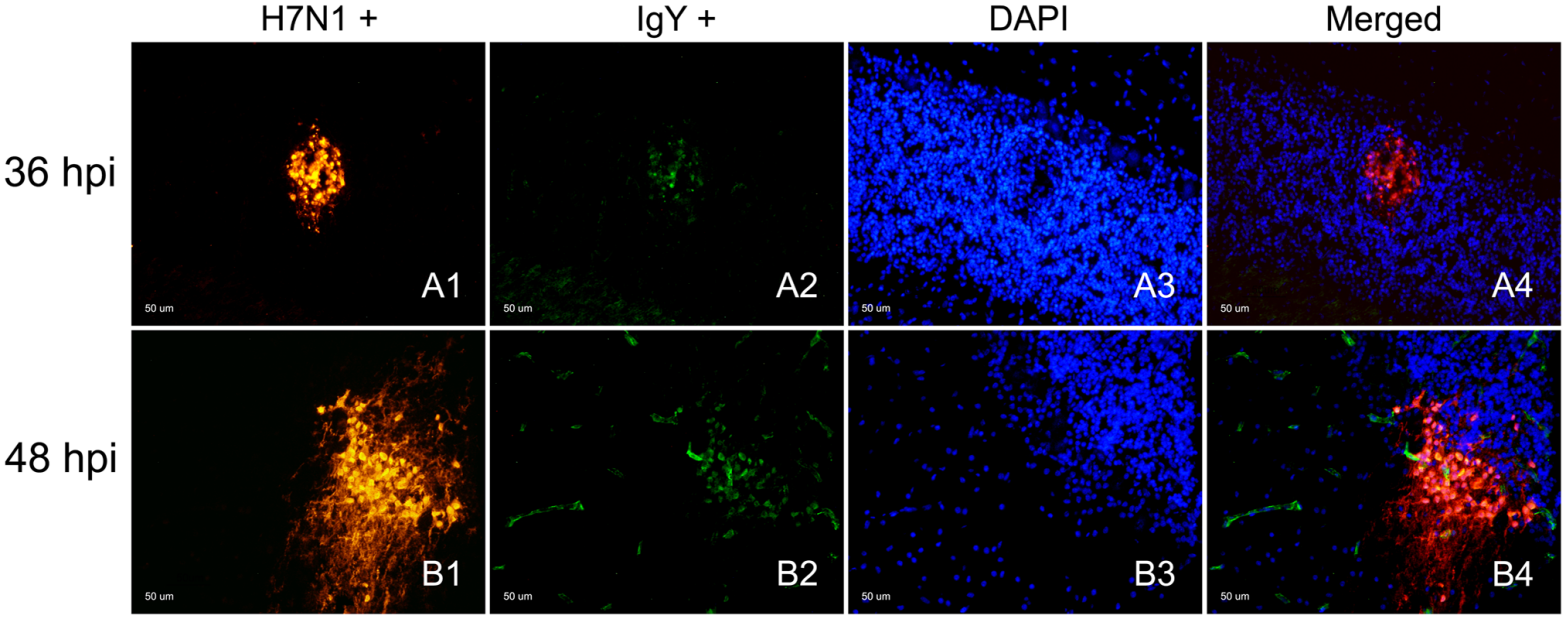
Detection of IAV and IgY leakage in fresh frozen samples of H7N1 infected chickens. Immunofluorescence staining to detect IAV antigen and its association with IgY extravasation in chickens infected at 36 and 48 hpi, 50 µm. Influenza viral antigen was found in the cerebellum focally infecting some blood vessel cells and a group of neural cells between the Purkinje and granular layer of the cerebellum (Cb) (A1, B1) at 36 and 48 hpi, but with evident increase in intensity at 48 hpi (B1). IgY extravasation was found in the same focus (A2, B2), staining the neuropil and brain cells surrounding the affected vessel. The superimposed image (A4, B4) showed that the IgY extravasation foci corresponded to an area where there was influenza viral antigen.

Colocalization of IgY extravasation zones and IAV-positive zones was observed in infected chickens at 36 and 48 hpi (Fig. 4). This codetection was more evident in sites where the IgY leakage formed large foci (Fig. 4, A4–B4). In general, the diameter of the IgY extravasation staining was less intense, but equal in extent with respect to the diameter of the IAV antigen-positive foci.

### Pattern of staining for the tight junction proteins claudin-1 and ZO-1 in the brain

**Figure 5:**
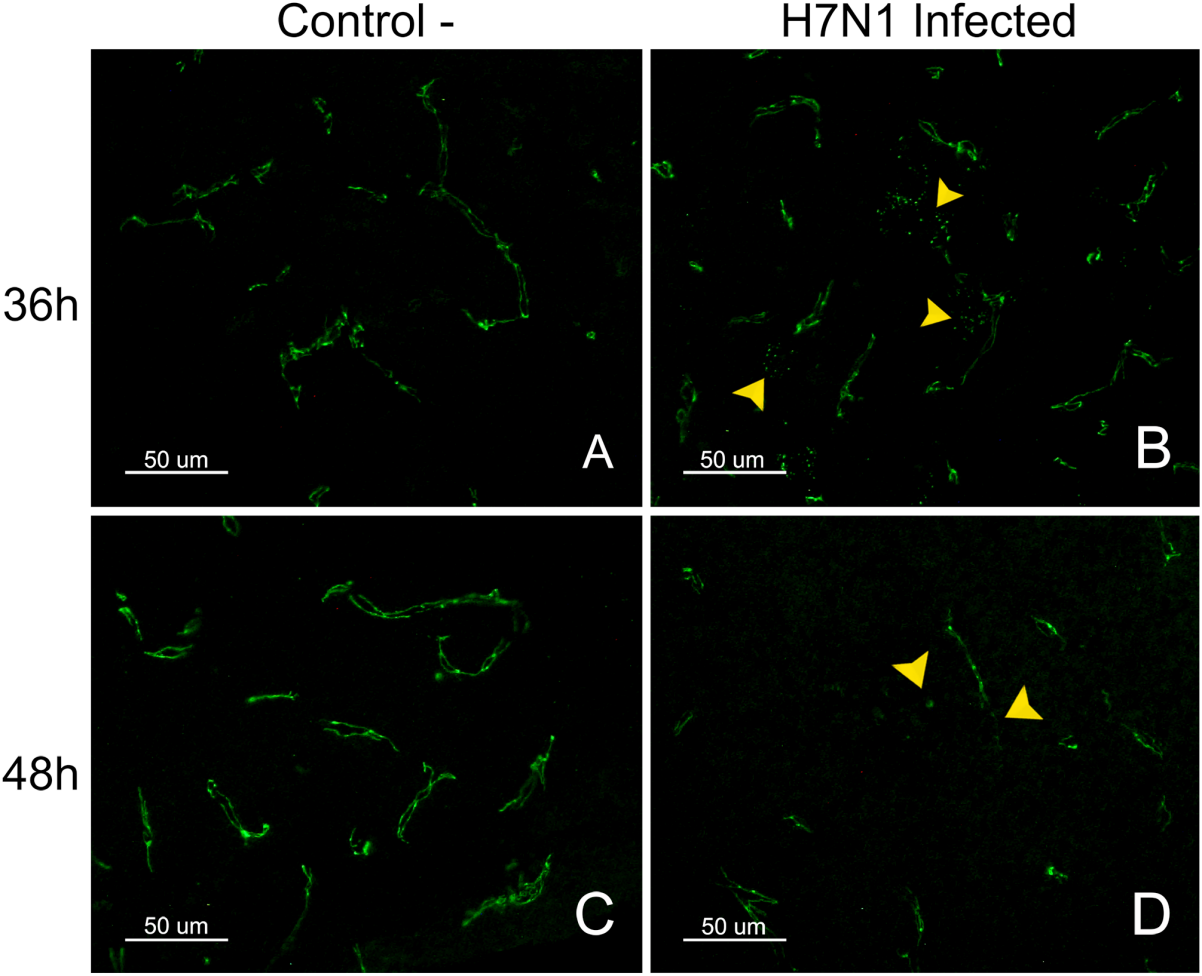
Pattern of staining for the TJ protein ZO-1 in control and H7N1 infected chickens. ZO-1 staining in the telencephalic pallium (Pall) of control chickens was observed as a continuous line in both sides of the blood vessels (A, C). In contrast, a granular and discontinuous appearance of the blood vessels was observed in the brain of infected chickens at 36 hpi (D). The loss of ZO-1 was multifocal in the brain of chickens at 48 hpi (B) (50 µm).

ZO-1 staining was observed as a continuous line externally bordering the endothelial cells in the brain parenchyma, meninges, choroid plexus (Chp) and CVOs (AP and ME). In the arterioles, ZO-1 staining was observed in the intercellular junctions between the endothelial cells. ZO-1 staining was also found between epithelial cells of the Chp, where the protein outlined the perimeter of the cells, resulting in a honeycomb-like pattern. A similar pattern was observed in the CVOs, and the staining was denser than in the Chp. Claudin-1 staining was only observed in the CVOs (AP) and the choroid plexus, where it exhibited the same pattern as the ZO-1 staining. The TJ proteins were not detected between the ependymal cells. The intensity and pattern of claudin-1 staining were not different in control and infected chickens at any hpi. In contrast, the pattern of ZO-1 staining showed changes in infected chickens evaluated at 36 and 48 hpi in comparison with the negative controls. These alterations consisted mainly of focal loss of the characteristic structure of the blood vessels, which seemed discontinuous or granular in appearance. Moreover, at 48 hpi, there were foci where the ZO-1 staining was totally absent (Fig. 5, D). The pattern and intensity of staining for ZO-1 and claudin-1 in the CVOs and choroid plexus did not show any visible alterations in the infected animals at any hpi compared with non-infected controls.

### Codetection of ZO-1 and IAV antigen

**Figure 6:**
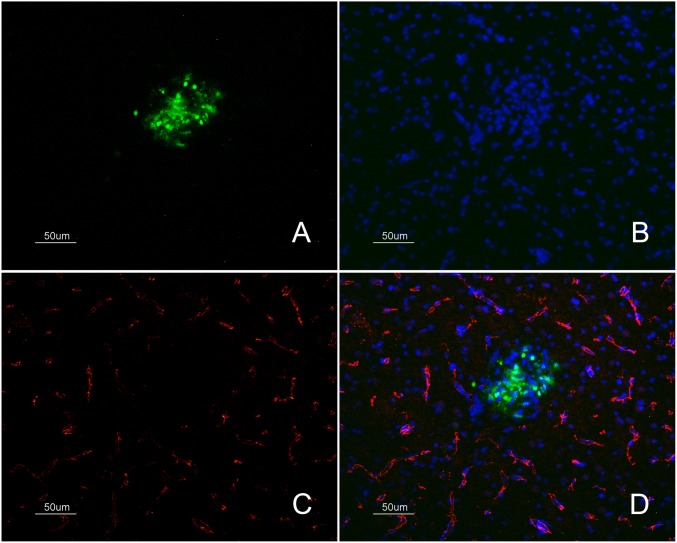
Detection of the TJ protein ZO-1 and IAV antigen in samples of H7N1 infected chickens. Immunofluorescense staining of fresh frozen brain samples of infected chickens at 36 hpi showing the loss of ZO-1 marker (C) (labelled in red colour) in a focus of gliosis (B) positive for influenza viral antigen (A) (labelled in green colour) found in the telencephalic pallium (Pall) (50 µm). Merged image showing the absent of ZO-1 marker labelling that is affecting specifically the area of gliosis where IAV antigen was found (D).

To confirm that the abnormal pattern of ZO-1 staining or its absence was the consequence of the direct replication of the H7N1 HPAI virus, double immunofluorescence labeling of ZO-1 and the IAV antigen was performed on brain samples from infected and control chickens at 18, 24, 36 and 48 hpi. This double staining confirmed that the loss of ZO-1 staining corresponded to areas where the viral antigen was present (Fig. 6).

### Detection and distribution pattern of viral antigen during the first hpi in formalin-fixed brain sections

**Figure 7:**
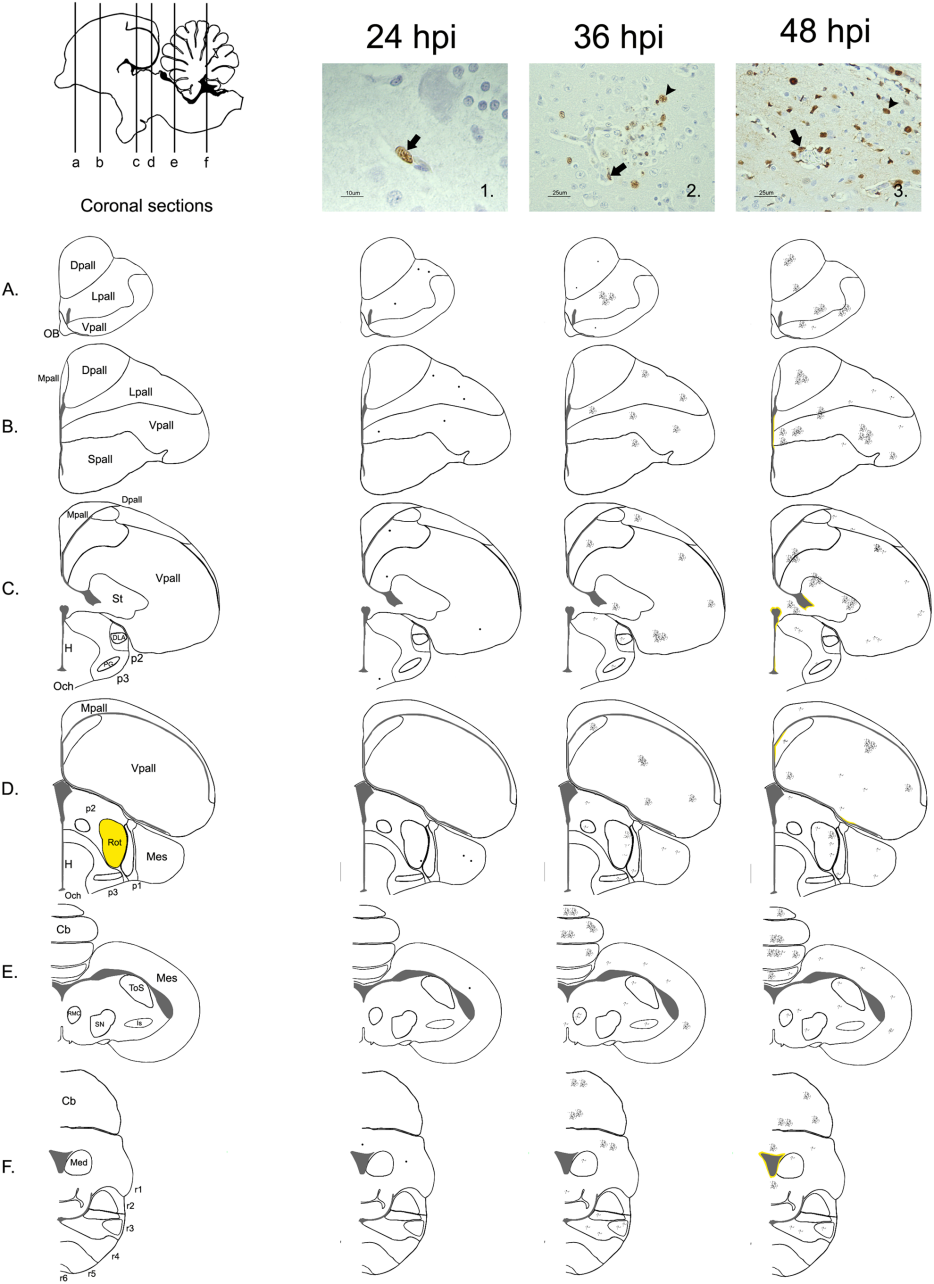
Distribution of the HPAI H7N1 antigen detected by immunohistochemistry in formalin fixed chicken’s brain samples. Schematic sagittal drawings of the chicken’s brain showing the distribution of the IAV antigen at 24, 36 and 48 hpi, according to the coronal levels represented in the diagrams below (A, B, C, D, E, F). In this study, the intensity of the staining was always scarce; consequently, in the diagram corresponding to 24 hpi, a dot was drawn in those regions where a positive cell was found. Microphotography 1. shows an endothelial cell (black arrow) with positive viral antigen staining in the nucleus (10 µm). At 36 and 48 hpi, the intensity of influenza virus antigen staining was slight, varying the number of cell from 2 to 80 positive cells per foci. Bilateral staining was only found in the Rot (thalamus- p2) at these hours (labelled in yellow in the diagram of the left). Microphotography 2. shows a neuron surrounded by several positive glial cells (arrowhead) beside a disrupted capillary (black arrow) at 36 hpi, located in the Rot (p2) (25 µm). Positive viral antigen was detected in few ependymal cells until 48 hpi. Microphotography 3. shows the presence of viral antigen in endothelial cells (black arrow), glial cells (arrowhead), neurons, and ependymal cells (white arrow) (25 µm).

Viral antigen was detected in formalin fixed tissues at 24, 36 and 48 hpi. At 24 hpi, the viral antigen immunolabeling was scarce and restricted to the nucleus of a few individual endothelial cells in the telencephalic pallium (Lpall and Vpall), mesencephalon and cerebellum of one chicken (Fig. 7). At 36 and 48 hpi, multiple foci consisting of viral antigen-positive endothelial cells, glial cells and neurons were found mainly in the telencephalic pallium (Dpall, Lpall, Vpall) and the cerebellum. Viral antigen was also observed in the subpallidum, prethalamus, thalamus, mesencephalon and rhombomeres 3 and 4. These foci were composed of a variable number of positive cells (5–70 cells), in which the viral antigen staining was mainly found in the nucleus of the cells and occasionally in the cytoplasm. At 48 hpi, there were also a few foci where the neuropil showed positive granular staining. In general, the intensity of viral antigen-positive staining was higher in the intermediate area of the telencephalon (Fig. 7, B–C) in comparison with the OB and most cranial areas of the brain. Viral antigen staining of ependymal cells was not detected until 48 hpi, and consisted of a line of positive cells (10–70 cells) focally in the lateral, third and fourth ventricles. As in the other cells of the brain, the viral antigen staining was mainly nuclear. No viral antigen was observed in the epithelial cells of the choroid plexus. Evidence of bilateral staining was only observed in the thalamus at 36 and 48 hpi and consisted of a scarce number of positive endothelial cells or neurons. None of the CVOs evaluated (AP and ME) showed IAV antigen staining at any time post-infection.

### Quantification of viral RNA in blood by RT-qPCR

**Figure 8:**
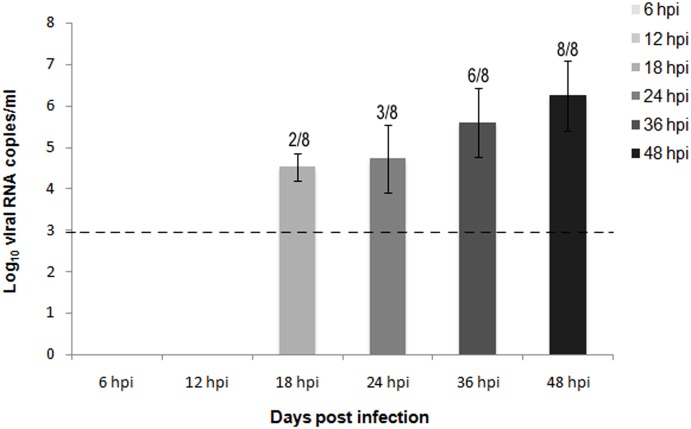
Quantification of viral RNA in blood samples of H7N1 inoculated chickens from 6 to 48 hpi. Viral RNA was detected by by quantitative real time RT-PCR (RT-qPCR) in blood samples of chickens infected with the HPAI virus H7N1 in increasing levels from 18 hpi to 48 hpi. Viral RNA levels were expressed as log10 viral RNA copies/µl. Limit of detection is indicated with the dashed line. The number of positive samples from the total number of animals is indicated above each bar.

Viral RNA in blood was first detected in 2 out of 8 chickens at 18 hpi (4.52 Log_10_ viral RNA copies/ml). A similar viral load was detected in 3 out of 8 chickens at 24 hpi (4.72 Log_10_ viral RNA copies/ml). Viral RNA levels increased at 36 hpi (5.60 Log_10_ viral RNA copies/ml), when they were detected in 6 out of 8 chickens. These levels peaked at 48 hpi (6.24 Log_10_ viral RNA copies/ml), when all of the sampled chickens showed high levels of viral RNA in blood (Fig. 8).

## Discussion

HPAI viruses produce viremia [32], [33] and lesions in the CNS of chickens [34] that hypothetically occur by disruption of the BBB [9], [35]. In general, the BBB can be disrupted by three mechanisms: the paracellular and transcellular routes, the “Trojan horse” mechanism and the leakage of infectious agents through disrupted endothelial cells [17], [36], [37]. In this study, SPF chickens infected with an H7N1 HPAI virus were examined during the first hpi to evaluate the integrity of the BBB and to determine whether the BBB is disrupted and how the infection of the central nervous system evolve during the first 48 hpi. We proceeded to use three different methods to qualitatively evaluate BBB permeability in chickens after inoculation with the aforementioned HPAI virus.

The first method used to evaluate the integrity of the BBB consisted of the intracardial perfusion of EB tracer at different hpi. This procedure has been frequently used on mice and rats to study different aspects of the pathogenesis of several infectious and non-infectious diseases that affect the CNS [38]. In contrast, this method has been scarcely used in chickens [39], [40] and, to our knowledge, it has not been used to study the integrity of the BBB in chickens exposed to infectious agents. In the current work, chickens perfused with EB fluorescence tracer every 6 hpi demonstrated extravasation until 48 hpi. The most frequent zones where EB diffused from the circulating blood into the parenchyma were the telencephalic pallium and the cerebellum, followed by the thalamus (Rot), subpallium (Spall) and brainstem. EB leakage was observed as multiple foci, indicating that the loss of BBB function occurs simultaneously in multiple areas of the brain. Additionally, we were able to demonstrate the presence of viral antigen in some of these areas of extravasation, suggesting that the EB leakage may be caused by the direct replication of the virus in brain cells. Evidence of the affinity of the virus for the telencephalic pallium (Pall) and the cerebellum (Cb) was also confirmed by conventional IHC, which showed that the viral antigen-positive areas were mainly located in these same regions from 24 to 48 hpi.

Regarding the distribution of the areas of EB extravasation and the presence of viral antigen, we observed that the lesions and topographical distribution of the viral antigen tended to be allocated bilaterally and symmetrically. This differential distribution is in agreement with previous studies, in which three different HPAI viruses showed similar tropism in the brain (A/chicken/Victoria/1/85 (H7N7), A/turkey/England/50-92/91 (H5N1), A/tern/South Africa/61 (H5N3)) [35]. This pattern was observed in the diencephalon, mesencephalon, and rhombencephalon, especially in the *Rotudus* (thalamus) (p2), PG (p3), ToS (mesencephalon), and APT nuclei (p1). Moreover, it was especially remarkable that the viral antigen began to show preference for the thalamus during the first hpi. This was significant considering that, in humans, IAV causes a series of CNS disorders and particularly for ANE, bilateral and symmetrical lesions have also been described in the thalamus, putamen, cerebral and cerebellar white matter and brainstem tegmentum [12], [41], [42], [43]. For this reason, and because it is suggested that ANE is also caused by a disruption of the BBB, the chicken model has been proposed for studies to help understand this mechanism of pathogenesis [9]. Moreover, the preferential targeting of the thalamus is also common to other infectious agents, such as rabies [44], herpes viruses [45], flaviviruses (Japanese Encephalitis, West Nile Encephalitis and Murray Valley Encephalitis) [46] **,** measles [47] and HIV [48]. However, in contrast to the chicken model, viral antigen and RNA are rarely detected in the brain tissue and CSF of patients with ANE [49], [50], [51]. Hence, both diseases seem to be induced by different mechanisms. In chickens, the lesions are caused by direct replication of the virus, while in humans, it is suggested that immune-mediated mechanisms are involved [52], [53]. The affinity of the virus for these regions together with the increased extravasation of EB in these areas could indicate that the BBB response may be different in each brain region, as is suggested to occur in other pathological insults [54], [55].

With the second method, the integrity of the BBB was evaluated by detecting an increased vascular permeability of plasma proteins (IgY) at different hpi. In this study, an extravascular distribution of the endogenous serum protein IgY was demonstrated at 36 and 48 hpi. The deposition of IgY in the brain parenchyma increased in extension along the time, indicating a progressive increase in the severity of the BBB damage after infection. IgY extravasation areas coincided with EB leakage zones. However, the IgY staining was less intense and widespread than the EB staining. This discrepancy in the intensity of staining and the sensitivity of detection of extravasation areas between both techniques may be related to differences in the sensitivity detection of both markers and in the molecular weight of the two molecules, which determines their penetration. That is, extravasated IgY was detected using a less sensitive direct IF technique, resulting in a weak staining [56], whereas the EB perfusion technique made it possible to fix the sample at the same time that the animal was perfused, resulting in intense labeling in the zones of leakage [23]. Moreover, the BBB does not allow the passage of soluble molecules greater than 400 Da [57]. Thus, the size of the molecules that are used as tracers may also determine the ability to detect minor changes in the permeability of the BBB. As a result, the IgY molecule (165000 Da), due to its large size [58], diffuses only when the damage to the BBB is severe, and the molecule spreads more slowly after extravasation than smaller molecules would. Most of the IgY remains trapped in the brain parenchyma close to the disrupted BBB area [59]. For this reason, the IgY molecule was restricted to areas where viral antigen was present, indicating direct damage of the vessels and the nearest cells. In contrast, the EB molecule (960.81 Da), which binds to albumin (69000 Da), can spread more easily from the point of disruption in the BBB to the parenchyma owing to its smaller size, which explained why it was more widely distributed than IgY protein and why it was found in areas where viral antigen was not detected. Staining for both EB tracer and IgY were found in the cytoplasm and nucleus of the cells in the damaged areas, which is in agreement with previous experiments were neuronal uptake of EB was related to the presence of extravasation and injury to the CNS [23], [60]. Similarly, the extravasation of plasma proteins, such as IgY, and their accumulation in neural cells has been related to permanent cell injury in the affected cells [14], [59], [61], as was also observed in this study.

Finally, the third method consisted of the evaluation of the integrity of the BBB using the ZO-1 marker, which is a reliable indicator of the presence of undamaged TJs between the endothelial cells of the BBB [62]. This marker enabled us to determine whether the virus was able to enter the brain by disrupting BBB TJs or by using transcellular or paracellular routes, as has been observed with other infectious agents. One such example is the West Nile virus, which enters the brain without impairing the integrity of BBB TJs [19], [63]. On the other hand, we observed disorganization and loss of the continuous linear arrangement characteristic of ZO-1 in the brains of infected chickens at 36 and 48 hpi. This pattern was observed in multiple foci in the telencephalon, diencephalon, mesencephalon and cerebellum. Moreover, there were focal areas where the ZO-1 marker was absent, suggesting that the BBB had been completely disrupted. The loss or dissociation of ZO-1 from the junctional complexes is associated with increased barrier permeability [64]. Similarly, decreased intensity of ZO-1 immunolabeling has been observed in mouse and rat models of different pathological brain conditions, such as ischemia [65], traumatic injury [66], stroke [67], human encephalitis caused by HIV [54] and in *in* *vitro* and *in* *vivo* models of HIV [68], SIV [69], and human T-cell leukemia virus [70]. Codetection of ZO-1 and IAV antigen permitted confirmation that the core of the areas exhibiting loss of ZO-1 labeling corresponded with viral antigen-positive areas. This finding agrees with previous *in* *vivo* and *in* *vitro* studies that demonstrated loss of ZO-1 in the brains of mice infected with influenza (A/WSN/33) and in polarized epithelial cells infected with influenza virus [71], respectively.

Previous studies indicated that the TJ proteins claudin-1, claudin-5, occludin and ZO-1 can be found in chicken endothelial cells [72]. However, in this study, claudin-1 was only found between the epithelial cells of the Chp and CVOs, and claudin-1 labeling did not vary between infected and non-infected chickens. This difference can be attributed to the confounded data generated by the use of antibodies that cross-react with claudin-3 in previous publications [72], [73], [74].

In a previous study from our group, IAV was found in endothelial cells and the epithelium of the Chp, suggesting that Chp could be the initial sites of infection in the brain. Other infectious agents use the Chp and CVOs, which form the blood cerebrospinal fluid barrier (BCSFB), to enter the CNS. These tissues have a fenestrated endothelium and a lower electrical resistance than the BBB. For example, the Chp is a portal of entry for canine distemper virus (CDV) [75], lymphocytic choriomeningitis virus (LCMV) [76], feline immunodeficiency virus (FIV) [77] and HIV [78]. Likewise, the CVOs may be a route of entry for *Trypanosoma brucei* parasites [79], and scrapie and BSE in sheep and goats [80]. In contrast, no alterations in the pattern and intensity of ZO-1 and claudin-1 staining were observed in the epithelial cells forming the Chp or in the tanycites (specialized ependymal cells of the CVOs) of infected chickens at any hpi. Therefore, the evidence provided by this study indicates that the BCSFB does not contribute to the initial invasion of this HPAI virus H7N1 into the CNS.

Taken together, our observations demonstrate the chronological sequence of invasion of the H7N1 HPAI virus in the CNS of chickens. We determined that soon after inoculation, the H7N1 HPAI virus invades the bloodstream (18 hpi), first infecting and replicating in blood vessels cells (24 hpi) and later producing disruption of the TJs, which was demonstrated by the loss of ZO-1 in the BBB (36–48 hpi). Simultaneous to the disruption, serum proteins leak from the bloodstream toward the tissue (IgY leakage), and the virus appears in brain parenchymal cells (neurons and glial cells) (36–48 hpi). The mechanism of BBB destruction and loss of ZO-1 has been studied in mice, in which it has been associated with upregulation of several cytokines, such as tumor necrosis factor (TNF)-α, interleukin (IL)-1 and IL-6. These cytokines participate in the induction of trypsin, which mobilizes calcium toward the cell and also induce the conversion of the non-active form of matrix metalloprotease (MMP) 9 into the active MMP-9 form. Both result in damage of the basement membrane and TJs disruption, including ZO-1 [71]. However, the molecular mechanism behind the disruption of the TJs in endothelial cells has not been completely elucidated, neither was covered in this study. Therefore, future studies should be directed to understanding the mechanism behind the disruption of the TJs. Besides, additional experiments using other highly pathogenic influenza viruses might be of value to determine if they follow the same sequence and mechanism of entry into the CNS.
